# Assessing the effectiveness of targeted educational interventions on enhancing self-efficacy and foot care practices among diabetic women in Jordan

**DOI:** 10.3389/fpubh.2024.1502781

**Published:** 2025-01-07

**Authors:** Heba Hijazi, Rabah Al Abdi, Sawsan Abuhammad, Wegdan Bani Issa, Alham Al-Sharman, Nesreen Saadeh, Alounoud AlMarzooqi, Fatma Refaat Ahmed, Ahmed Hossain, Hadia Radwan, Muhammad Arsyad Subu, Mohamad Alameddine

**Affiliations:** ^1^Department of Health Care Management, College of Health Sciences, University of Sharjah, Sharjah, United Arab Emirates; ^2^Department of Health Management and Policy, Faculty of Medicine, Jordan University of Science and Technology, Irbid, Jordan; ^3^Department of Electrical, Computer, and Biomedical Engineering, College of Engineering Abu Dhabi University, Abu Dhabi, United Arab Emirates; ^4^Department of Biomedical Engineering, Faculty of Engineering, Jordan University of Science and Technology, Irbid, Jordan; ^5^Nursing Department, College of Health Sciences, University of Sharjah, Sharjah, United Arab Emirates; ^6^Department of Maternal and Child Health, Faculty of Nursing, Jordan University of Science and Technology, Irbid, Jordan; ^7^Department of Physiotherapy, College of Health Sciences, University of Sharjah, Sharjah, United Arab Emirates; ^8^Rehabilitation Sciences Department, Faculty of Applied Medical Sciences, Jordan University of Science and Technology, Irbid, Jordan; ^9^Department of Internal Medicine, Faculty of Medicine, Jordan University of Science and Technology, Irbid, Jordan; ^10^Critical Care and Emergency Nursing Department, Faculty of Nursing, Alexandria University, Alexandria, Egypt; ^11^Department of Clinical Nutrition, College of Health Sciences, University of Sharjah, Sharjah, United Arab Emirates

**Keywords:** diabetic foot, foot care practice, self-efficacy, diabetic women, foot ulcers

## Abstract

**Background:**

Diabetic foot is a major public health issue, leading to increased morbidity and mortality among diabetic patients. This study aimed to evaluate the effectiveness of targeted health education interventions on self-efficacy and foot care practices among diabetic women in Jordan.

**Methods:**

A pretest-posttest, quasi-experimental design was used to collect data from 76 diabetic women at a tertiary hospital in northern Jordan. Participants were assigned to three groups: a control group receiving standard care; Intervention Group 1, receiving standard care with weekly text reminders and follow-up calls; and Intervention Group 2, receiving the same components as Intervention Group 1, plus small group education sessions and hands-on foot care training. Generalized Estimating Equations models were used to assess the impact of the interventions on foot care practices and self-efficacy over an 8-week period.

**Results:**

The findings revealed that participants in Intervention Group 2 demonstrated the most significant improvements in both foot care practices and self-efficacy. For foot care practices, Intervention Group 2 had adjusted odds ratios (aORs) of 2.5 (95% CI: 1.3–5.1) and 1.7 (95% CI: 1.2–2.9) when compared to the control group and Intervention Group 1, respectively. Similarly, for self-efficacy, the aORs for Intervention Group 2 were 2.7 (95% CI: 1.4–5.2) relative to the control group, and 1.8 (95% CI: 1.1–3.2) compared to Intervention Group 1.

**Conclusion:**

Our study demonstrates that interactive educational approaches—featuring group discussions, real-time problem-solving, immediate feedback, and family support—can empower diabetic women to take a more active role in managing their foot health. Routine clinical care alone is insufficient to promote proactive foot care behaviors, highlighting the need for healthcare providers to incorporate educational materials tailored to the local cultural context into standard care to enhance patient outcomes.

## Introduction

Diabetes-related foot disease (DFD) is a complex and debilitating condition often associated with type 2 diabetes mellitus (T2DM), encompassing a spectrum of complications that significantly impact patient health and quality of life (1, 2). DFD is generally characterized by the formation of diabetic foot ulcers (DFUs) or foot infections in individuals with diabetes, often associated with peripheral neuropathy (PN) or peripheral artery disease (PAD) (3). The PN results from nerve damage caused by high blood sugar levels, leading to loss of sensation in the feet (3). However, PAD involves reduced blood flow to the lower limbs due to narrowing or blockages in the arteries, increasing the risk of foot ulcers and infections (4, 5).

Of the worldwide population of 537 million diabetic adults aged 20–79 years, an estimated one-third are at risk of developing DFD (6), with 20 million currently affected by DFD, and up to 2 million requiring amputation annually (5). Eventually, one in two poorly healed cases progresses to infectious DFD, potentially leading to lower extremity amputations (7). Amputation incidence is commonly used as a key indicator of the burden associated with DFD (8, 9), which is a primary cause of hospital admissions among diabetics (10). According to recent systematic reviews and meta-analyses, several studies have revealed that DFU and amputation have been also linked to elevated mortality rates among diabetic patients (6, 11).

Alarmingly, over 75% of adults with diabetes live in low- and middle-income countries, where prevalence is increasing more rapidly than high income countries (12). In Jordan, the burden of diabetes and its associated foot complications is rapidly escalating, where high rates of T2DM have been observed over the past few decades (13). The prevalence of diabetes among men aged ≥25 years has increased from 14.2% in 1994 to 18.3% in 2004, 26.8% in 2009, and 32.4% in 2017 (14). For women, the corresponding prevalence rates were 12.3, 16.9, 18.8, and 18.1%, respectively (14). Additionally, the foot at risk is prevalent in 17.2% of diabetic patients in the country (15), with DFU estimated at 5.3% (16).

Empirical evidence indicates that poorly managed DFD contributes to sensation loss, chronic pain, discomfort, and high healthcare costs due to ongoing treatments and complications (7). Patients may also experience emotional challenges, such as limited mobility, reduced work capacity, and compromised quality of life, which can lead to anxiety, depression, and other mental health issues (17–19). However, early detection of foot risk and preventive care can significantly reduce DFD complications (20, 21). The National Institute for Health and Care Excellence (NICE) guidelines recommend regular foot examinations for all adults with diabetes to prevent complications through proper care and timely intervention (22). Despite this, many diabetic individuals, especially women, do not undergo foot examinations in healthcare settings (19, 23). Targeted educational interventions through alternative methods, such as virtual delivery, can improve outreach, participation, and effectiveness for diabetic women by overcoming barriers like geographic limitations, cultural restrictions, and time constraints.

Research highlights gender-specific factors affecting women’s foot health, including hormonal changes, footwear choices, and obesity, which contribute to conditions such as plantar fasciitis, foot deformities, and heel spurs (2, 24–29). Women with diabetes are at higher risk of PN, which increases the likelihood of ulcers, infections, and reduced bone density, potentially leading to stress fractures (29, 30). Furthermore, women are more susceptible to varicose veins and venous insufficiency, which can cause swelling, pain, and other symptoms that exacerbate foot problems and reduce mobility (31).

Considering these potential complications, it is crucial to understand the level of foot self-care among diabetic women to guide early supportive strategies and promote effective management. To address this need, our study aimed to investigate the impact of a targeted educational intervention on enhancing self-efficacy and improving foot care behaviors among women with T2DM in northern Jordan.

### Study framework

**Figure 1:**
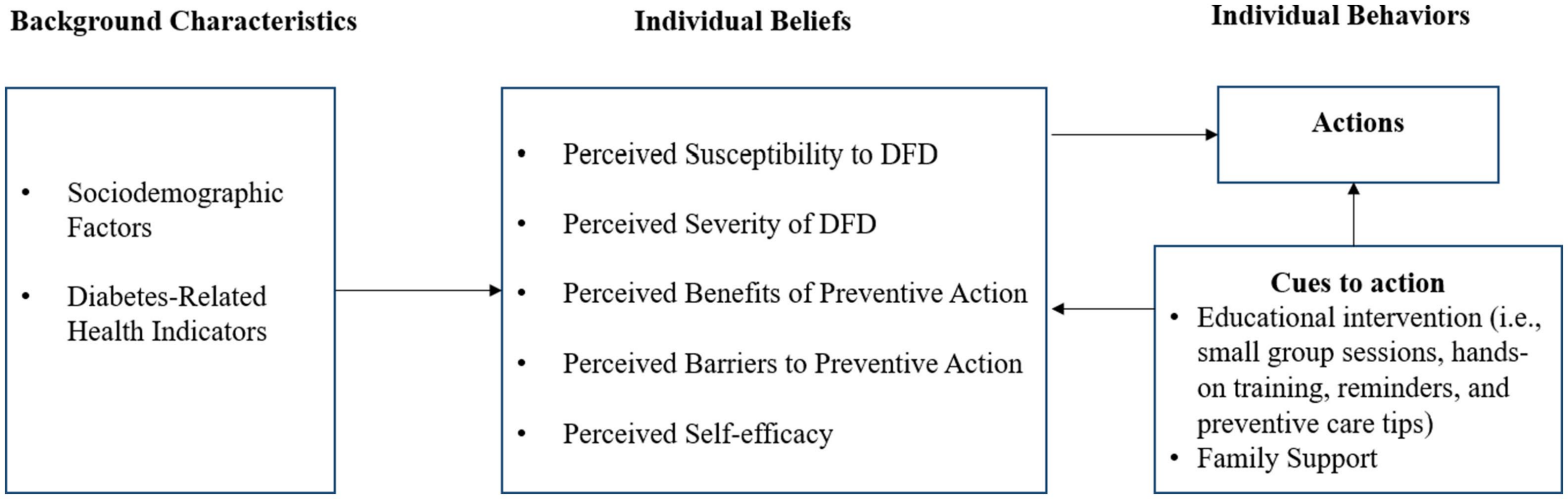
Framework for evaluating the impact of targeted educational interventions on foot self-care in diabetic women. DFD, Diabetes-related foot disease. Adapted from health belief model by Becker (32).

Our study utilized the Health Belief Model (HBM) to guide the development of a framework for an effective educational intervention, emphasizing DFD prevention and the adoption of foot self-care behaviors. The HBM has been widely applied in public health to design and evaluate health education and behavior change interventions structured to address these key components such as perceived susceptibility, severity, benefits, and barriers, as well as cues to action and self-efficacy (32–35). The theoretical framework for foot self-care behavior is depicted in Figure 1.

Aligned with the HBM, our framework suggests that patients will take steps to prevent DFD if they perceive themselves as vulnerable to foot-related risks (perceived susceptibility). Therefore, it is essential to inform patients of their personal risk of developing diabetic foot complications and the criteria that classify them as low, moderate, or high risk. This awareness (cues to action) encourages preventive actions, such as regular foot exams, proper foot care education, and timely reporting of foot issues (7, 36).

Since managing DFD relies heavily on patient involvement, educational interventions should focus on ensuring that patients understand the negative consequences of neglecting diabetic foot care, including risks like foot ulcers, infections, and potential amputations (perceived severity) (37). This awareness underscores the benefits of proactive foot care in reducing healthcare costs, enhancing mobility, increasing independence, and improving overall quality of life (perceived benefits) (17, 18).

Recognizing that many patients face barriers to effective foot care, educational interventions must address the primary obstacles to adopting actionable steps (perceived barriers). These barriers include limited awareness, forgetfulness, lack of motivation, discomfort with foot inspections, competing priorities, and inadequate access to resources (38). Providing comprehensive foot care education that fosters self-efficacy can help patients overcome these challenges, adhere to preventive practices, and enhance their ability to adopt and maintain healthy foot care behaviors (39).

## Materials and methods

### Study design and setting

Using a pretest-posttest, quasi-experimental design, data was collected from diabetic women who attended Internal Medicine, Endocrinology, and Diabetes clinics at a leading tertiary referral hospital in northern Jordan.

### Data collection procedure

A nonprobability convenience sampling method was utilized to recruit study participants who came for follow-up treatment at the identified clinics. Prior to data collection, a nurse at each clinic conducted a screening to determine participant eligibility. The inclusion criteria included adult females diagnosed with T2DM who regularly attended the clinic at the specified hospital, were willing to participate, were reachable by telephone, could read and understand educational materials, and were at low risk of developing DFD. Based on NICE guidelines, a widely accepted categorization of DFD risk in the literature, patients were categorized as low risk if they had no evidence of PN, PAD, foot deformity, impairment, or previous ulcers or amputation (19, 22, 40). By targeting a low-risk sample, the study ensures a homogeneous group for assessing the effectiveness of the targeted educational interventions, providing more time to improve foot care. In contrast, moderate or high-risk patients would require more immediate medical interventions, which limits the preventive impact.

Exclusion criteria included adult diabetic women at moderate risk (such as those with foot deformity, PN, or non-critical limb ischemia) or high risk (such as those with a history of previous ulceration, amputation, or more than two of the following: PN, PAD, or deformity) for developing DFD, according to NICE guidelines. Additionally, individuals with cognitive impairments or mental health conditions affecting their comprehension of educational material were excluded. It is worth mentioning that the categorization of DFD risk was further assessed by resident doctors at the clinics, following established recommendations (41). The evaluation involved examining various skin conditions, including dryness, cracked skin, fungal infections (characterized by itching and scaling), corns and calluses caused by friction, redness from sunburn or irritation, blisters due to ill-fitting shoes, and minor skin lesions.

The recruitment process was initiated by voluntarily inviting eligible patients to participate. Those who agreed were then asked to sign an informed consent form after receiving an explanation of their involvement and the study’s objectives. All participants were alternately assigned to either the control group, Intervention Group 1, or Intervention Group 2. This method ensured a balanced distribution of potential confounding variables across all groups.

To determine the sample size, a power analysis was conducted using G*Power software (42). With an anticipated medium effect size (Cohen’s *d* = 0.50), a power level of 0.80, and an alpha level of 0.05, the required sample size for a between-group comparison was estimated to be 66 participants. The medium effect size was chosen as it reflects a moderate impact of the intervention, which is a reasonable expectation for most behavioral or educational interventions (43).

**Figure 2:**
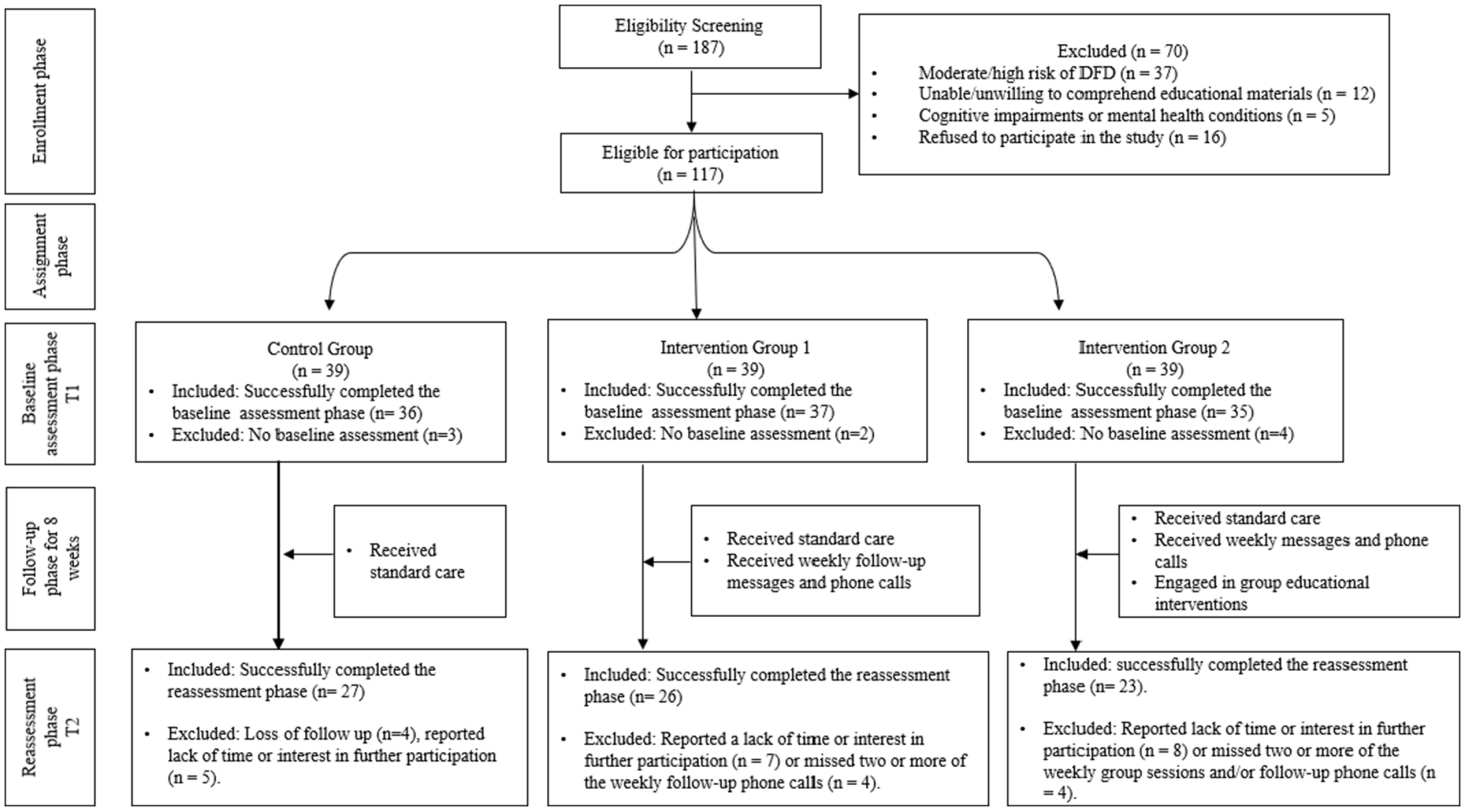
Outline of the patient flow from baseline to 8-week follow-up.

In our study, 108 participants successfully completed the baseline assessment, and 76 completed the reassessment phase following the 8-week intervention period. Our final sample of 76 participants exceeds this minimum requirement, providing adequate power to detect meaningful differences between study groups. This sample size also accounts for potential attrition—common in longitudinal studies—where researchers recommend increasing the sample size by 10–20% to ensure sufficient data for analysis (44). Figure 2 provides a detailed breakdown of participation rates throughout the study.

### Study groups

#### Control group

Patients in the control group received standard care, which included routine check-ups and monitoring of health parameters such as blood glucose levels [e.g., Fasting Blood Glucose (FBG) and glycated hemoglobin (HbA1C)], lipid profiles, and blood pressure. Additionally, they were given a brochure on foot care routines, covering topics such as proper footwear, nail care, and daily foot inspection, as well as a foot care kit containing diabetic socks, a nail clipper, and moisturizing cream.

This design was chosen to reflect the standard care provided in the local clinical setting, ensuring a practical and relevant baseline for comparison with the intervention groups. Although the inclusion of educational brochures and a foot care kit may reduce the contrast with the intervention groups, the objective was to evaluate the additional benefits of the targeted interventions rather than compare them against no care.

#### Intervention group 1

To reinforce foot care routines over the 8-week period, Intervention Group 1 received the same standard care as the control group, along with additional contact from the research team. At the start of each week, participants received a text message containing reminders for diabetic foot care. The messages included tips on inspecting feet daily for cuts, blisters, or swelling; maintaining proper foot hygiene with mild soap and lukewarm water; wearing clean, dry socks and properly fitting shoes; trimming toenails carefully; and seeking medical attention if signs of infection appear. Each message was followed by a phone call later in the week to ensure participants implemented the tips and to address any questions or concerns.

#### Intervention group 2

Throughout the study period, participants in Intervention Group 2 received the same care as those in Intervention Group 1, with the addition of targeted foot care educational materials delivered through online meetings accessible via mobile devices. The educational content, guided by the HBM, was developed by various professionals in endocrinology and diabetic care, podiatry, nutrition, nursing, and physical therapy, who also determined the number and duration of the required sessions.

The intervention for this group consisted of eight 60-min virtual sessions held weekly, each covering key aspects of foot care and diabetes management. Session 1 provided an overview of diabetes, foot-related symptoms, common complications like foot ulcers, and preventive measures, including understanding personal risk (perceived susceptibility and severity). Session 2 focused on practical foot care techniques, such as daily inspections, proper footwear selection, and early detection of foot issues (perceived benefits and cues to action). Session 3 discussed the role of nutrition in foot health and blood sugar management (perceived benefits). Session 4 featured instructional videos on foot care routines and hygiene, encouraging participants to practice these skills at home (self-efficacy).

Involving family members was also a key component in the later sessions to enhance support for foot care management. Accordingly, Session 5 focused on practical strategies for involving family in daily foot care routines and recognizing signs of foot complications, while also addressing challenges and reinforcing supportive behaviors (perceived barriers and cues to action). Session 6 expanded on family support by exploring ways to encourage medication adherence, promote a healthy diet beneficial for foot health, and recognize symptoms of hypo- and hyperglycemia that affect foot care (perceived benefits and self-efficacy).

Sessions 7 and 8 addressed broader aspects of foot care. Session 7 tackled the psychological aspects of being at risk for developing DFD (perceived susceptibility and perceived severity), focusing on stress management techniques, coping strategies, and the importance of mental health in foot care management (perceived benefits). Session 8 provided a comprehensive review of all topics covered, reinforcing key messages, and addressing any remaining questions (perceived barriers and cues to action).

Throughout the 8 sessions, interactive Q&A, group discussions, and practical demonstrations via video tutorials were used to ensure participants understood and could effectively apply the content. At the end of each session, the educational content was summarized in digital pamphlets, and key points were reviewed at the start of the following session to reinforce learning and address any concerns or inquiries.

### Variables and measurements

#### Baseline characteristics

Baseline data collected included patients’ sociodemographic variables (age, education, income, residence, occupation, marital status), lifestyle factors (smoking status), anthropometric measurements (height and weight), and self-assessed health status. The hospital’s electronic medical records were accessed to obtain details on diabetes-related health indicators, including recent FBG and HbA1C levels, diabetes duration, and comorbidities such as retinopathy, renal impairment, hypertension, anemia, and limited joint mobility. Healthcare utilization data, including health insurance coverage, hospitalization frequency, and follow-up clinic visits over the past year, were also collected.

### Outcome measures

To evaluate the intervention’s effectiveness on changes in foot self-care behavior across the study’s three groups, we assessed participants’ baseline and post-intervention foot care practices using the Diabetic Foot Self-Care Questionnaire (DFSQ) (45). The DFSQ is a 16-item tool measuring personal self-care (items 1–7), podiatric care (items 8–11), and appropriate use of footwear and socks (items 12–16), with each item scored on a five-point scale. Higher scores indicate better self-care and proactive foot care behavior. The DFSQ, originally developed for a Spanish population, has been validated in several languages, including Arabic, French, Persian, and Italian (46–49). The Arabic version of the DFSQ has demonstrated robust validity and repeatability among Arabic-speaking populations (49).

Additionally, foot care self-efficacy was measured before and after the intervention using the Foot Care Confidence Scale (FCCS) (50). The FCCS consists of 12 items rated on a five-point Likert scale, assessing confidence in performing foot care tasks such as cutting nails, washing feet, and purchasing appropriate footwear. Scores range from 12 to 60, with higher scores indicating greater self-efficacy. The FCCS has been validated in Arabic, demonstrating good internal consistency and reliability for assessing foot care confidence among Jordanian populations (51).

### Data analysis

Data analysis was conducted using IBM SPSS version 23. Descriptive statistics were used to summarize participants’ sociodemographic variables, diabetes-related health factors, and outcomes. Bivariate statistical tests, including t-tests for continuous variables and chi-square statistics for categorical variables, were used to compare data between the study groups. Fisher’s exact test was also applied when any category had fewer than 5 observations. The Wilcoxon Signed-Rank Test was applied to assess changes in self-efficacy and foot care practices within the same group before and after the intervention. Generalized Estimating Equations (GEE) were used to evaluate interactions between groups and time, adjusting for participants’ characteristics. This method accounts for the correlation of repeated measures within subjects and provides robust estimates of intervention effects (52). A *p*-value of <0.05 was considered statistically significant.

### Ethical statement

The study protocol was approved by the Institutional Review Board (IRB) of the Jordan University of Science and Technology and the Ethics Committee at the King Abdullah University Hospital (KAUH) (#40/140/2021). Informed consent was obtained from all participants, ensuring their voluntary participation. Confidentiality was maintained by assigning unique identification codes to each participant, with all data securely stored in password-protected electronic files accessible only to authorized researchers. Participant anonymity was further safeguarded by ensuring that no identifying information was included in the analysis or reporting. The study adhered to the ethical principles outlined in the Declaration of Helsinki.

## Results

### Participants’ characteristics

Table 1 presents the participants’ sociodemographic and diabetes-related health factors across three groups: the control group (*n* = 27), Intervention Group 1 (*n* = 26), and Intervention Group 2 (*n* = 23). The control group was significantly older (59.0 ± 4.4 years) compared to Intervention Group 1 (55.7 ± 6.4 years) and Intervention Group 2 (53.0 ± 7.4 years). Education levels also differed significantly, with Intervention Group 2 having the highest average (13.8 ± 2.8 years). Most participants in the total sample were married (72.4%), unemployed (40.8%), residing in urban areas (71.1%), had never smoked (60.5%), reported adequate financial resources (59.2%), and had health insurance (80.3%), with no significant differences between the groups for these variables.

**Table 1:** Participants’ characteristics by study (*n* = 76).

Variable	Control group (*n* = 27) *n* (%)	Intervention group 1 (*n* = 26) *n* (%)	Intervention group 2 (*n* = 23) *n* (%)	Total (*n* = 76) *n* (%)	*p*-value
Age in years (Mean, SD)	59.0 ± 4.4	55.7 ± 6.4	53.0 ± 7.4	56.5 ± 6.2	0.022
Education in years (Mean, SD)	12.3 ± 1.9	11.5 ± 2.4	13.8 ± 2.8	12.8 ± 2.4	0.043
Marital status					
Single/Widow/Divorced	5 (23.8%)	10 (47.6%)	6 (28.6%)	21 (27.6%)	0.263
Married	22 (40%)	16 (29.1%)	17 (30.9%)	55 (72.4%)	
Employment status					
Unemployed/Housewife	10 (33.3%)	10 (33.3%)	10 (33.4%)	30 (40.8%)	0.978
Retired	9 (34.6%)	9 (34.6%)	8 (30.8%)	26 (34.2%)	
Employed	8 (40%)	7 (35%)	5 (25%)	20 (25%)	
Area of residence					
Rural	8 (36.4%)	7 (31.8%)	7 (31.8%)	22 (28.9%)	0.960
Urban	19 (35.2%)	19 (35.2%)	16 (29.6%)	54 (71.1%)	
Smoking					
Never smoked	16 (34.8%)	13 (28.9%)	17 (37.8%)	46 (60.5%)	0.449
Former smoker	6 (42.9%)	5 (33.3%)	3 (20%)	14 (18.4%)	
Current smoker	5 (31.3%)	8 (50%)	3 (18.7%)	16 (21.1%)	
Income adequacy					
Inadequate	13 (41.9%)	10 (32.3%)	8 (25.8%)	31 (40.8%)	0.604
Adequate	14 (31.1%)	16 (35.6%)	15 (33.3%)	45 (59.2%)	
Health insurance					
No	5 (33.3%)	6 (40%)	4 (26.7%)	15 (19.7%)	0.866
Yes	22 (36.1%)	20 (32.8%)	19 (31%)	61 (80.3%)	
Self-perceived health status					
Poor	12 (50%)	7 (29.2%)	5 (20.8%)	24 (31.6%)	0.006
Average	6 (26.1%)	4 (17.4%)	13 (56.5%)	23 (30.3%)	
Excellent	9 (31%)	15 (51.7%)	5 (17.2%)	29 (38.1%)	
BMI					
<18.5 kg/m^2^	3 (42.9%)	2 (28.6%)	2 (28.5%)	7 (9.2%)	0.868
18.5 to <25 kg/m^2^	5 (26.3%)	6 (31.6%)	8 (42.1%)	19 (25%)	
25 to <30 kg/m^2^	11 (34.4%)	12 (37.5%)	9 (28.1%)	32 (42.1%)	
≥30 kg/m^2^	8 (40%)	6 (40%)	4 (20%)	18 (23.7%)	
Diabetes duration in years (mean, SD)	11.0 (8–14)	10.0 (7–12)	11.0 (8–13)	10.7 (8–13)	0.641
FBG (mmol/l) (mean, SD)	7.2 (6.5–8.0)	7.7 (6.7–8.2)	7.9 (6.8–8.4)	7.6 (6.7–8.2)	0.322
HbA1C (%) (mean, SD)	7.4 (6.9–8.1)	7.7 (7.2–8.3)	7.9 (7.3–8.5)	7.7 (7.2–8.3)	0.201
Presence of comorbidities					
None	10 (35.7%)	8 (28.6%)	10 (35.7%)	28 (36.8%)	0.629
1–2	11 (31.4%)	15 (42.9%)	9 (25.7%)	35 (46.1%)	
≥3	6 (46.2%)	3 (23%)	4 (30.8%)	13 (17.1%)	
Physician’s gender					
Male	21 (33.3%)	22 (35%)	20 (30.7%)	63 (82.9%)	0.663
Female	6 (46.2%)	4 (30.8%)	3 (23%)	13 (17.1%)	
No. of visits to the diabetes clinic per year					
1–3	17 (42.5%)	13 (32.5%)	10 (25%)	40 (52.7%)	0.392
4–5	7 (31.8%)	9 (40.9%)	6 (27.3%)	22 (28.9%)	
>5	3 (21.4%)	4 (28.6%)	7 (50%)	14 (18.4%)	
No. of hospitalization in the last year					
0	16 (37.2%)	15 (34.9%)	12 (27.9%)	43 (56.6%)	0.452
1–2	5 (22.7%)	9 (40.9%)	8 (36.4%)	22 (28.9%)	
≥3	6 (54.5%)	2 (18.2%)	3 (27.3%)	11 (14.5%)	

BMI, Body Mass Index; FBG, Fasting Blood Glucose; HbA1C, Glycated Hemoglobin; SD, Standard Deviation. Control Group, Patients who received standard clinical care; Intervention Group 1, Patients who received standard care supplemented with weekly text reminders and follow-up calls; Intervention Group 2, Patients who received all components of Intervention Group 1, plus small group education sessions and hands-on foot care training.

As detailed in Table 1, there were no significant differences between the three groups in Body Mass Index (BMI), diabetes duration, recent FBG and HbA1C levels, the presence of comorbidities, the gender of the primary supervising physician, or the number of clinic visits and hospitalizations over the past year (*p* > 0.05). In contrast, self-perceived health status varied significantly, with more participants in Intervention Group 1 rating their health as excellent (*p* = 0.006).

Participant attrition during the study was also assessed to evaluate its potential impact on the findings. Of the 108 participants who successfully completed the baseline assessment, 32 were excluded from the reassessment phase for reasons outlined in Figure 2. An analysis of characteristics showed no statistically significant differences between those who dropped out and the total sample. For instance, the mean age of dropouts was slightly higher compared to the total sample (57.2 ± 6.5 years vs. 56.5 ± 6.2 years, respectively; *p* = 0.48). Similarly, education levels did not differ significantly (dropouts: 12.5 ± 2.6 years vs. total sample: 12.8 ± 2.4 years, *p* = 0.52). Other variables, such as marital status (*p* = 0.61), employment status (*p* = 0.87), and self-perceived health status (*p* = 0.55), also showed no significant differences. These findings suggest that attrition did not result in systematic bias, supporting the robustness of the study’s conclusions.

### Comparative analysis of outcome measures within groups: baseline vs. follow-up

#### Changes in foot care practices

As shown in Table 2, personal self-care total scores improved significantly in both intervention groups after the 8-week follow-up. Median scores increased from 17.2 to 23.8 (*p* = 0.02) in Intervention Group 1 and from 16.5 to 25.9 (*p* = 0.001) in Intervention Group 2. However, the control group showed only a slight, non-statistically significant increase, with scores rising from 18.2 to 20.5 (*p* = 0.22). In the podiatric care subscale, Intervention Group 2 demonstrated significant improvement, with median scores increasing from 9.9 to 17.7 (*p* = 0.001). In contrast, neither the control group (10.5 to 11.6, *p* = 0.30) nor Intervention Group 1 (11.0 to 13.2, *p* = 0.12) showed significant changes.

**Table 2:** Median scores for foot care practices at baseline and 8-week follow-up within each group.

Outcome measures	Dimension/items	Control group		*p*-value^a^	Intervention group 1		*p*-value^a^	Intervention group 2		*p*-value^a^
		Baseline	Follow-up		Baseline	Follow-up		Baseline	Follow-up	
Foot care practices	Personal self-care	18.2	20.5	0.22	17.2	23.8	0.02*	16.5	25.9	0.001**
	1. Do you generally examine your foot yourself?	3.3	3.6	0.15	3.0	3.7	0.12	3.8	4.4	0.08
	2. Do you inspect your nails?	2.8	3.7	0.04*	3.7	4.1	0.18	3.5	4.3	0.15
	3. Do you look for sores and examine the state of the skin of your feet by yourself?	2.6	3.0	0.18	2.5	3.1	0.15	2.3	4.2	0.001**
	4. Is it hard for you to dry your feet after showering?	2.5	2.8	0.22	3.3	4.0	0.12	2.4	3.8	0.004**
	5. How often do you cut or treat your toenails?	2.9	3.5	0.02*	3.1	4.0	0.08	3.4	4.1	0.15
	6. Do you dry your feet?	3.3	3.7	0.19	3.4	4.6	0.03*	3.2	4.7	0.003**
	7. Do you heat your feet?	2.7	3.2	0.03*	2.6	4.1	0.03*	2.7	3.9	0.05
	Podiatric care	10.5	11.6	0.30	11.0	13.2	0.12	9.9	17.7	0.001**
	8. Do you treat skin sores, dry skin patches, and calluses?	2.9	3.1	0.26	2.7	3.6	0.20	2.8	3.5	0.10
	9. Regarding summer footwear, with excessive heat, do you take any precautions?	3.0	3.3	0.20	3.1	3.8	0.13	3.2	4.4	0.001**
	10. Regarding conventional footwear, do you check it before using it?	3.0	3.1	0.18	2.8	4.3	0.18	3.0	4.5	0.02*
	11. Regarding socks, do you select them with care?	2.9	3.0	0.24	3.2	4.0	0.08	2.8	4.2	0.03*
	Footwear and socks	12.6	13.1	0.19	13.2	16.0	0.25	11.3	23.2	0. 01*
	12. Regarding new shoes, do you take any specific steps before wearing them?	2.7	2.9	0.28	2.6	3.9	0.019*	2.9	3.8	0.15
	13. Is it hard to find comfortable shoes for your feet?	2.9	3.3	0.05	2.8	3.4	0.12	2.7	3.9	0.20
	14. Is it hard to find socks that are right for your feet?	2.8	3.0	0.18	3.4	3.9	0.12	2.9	4.0	0.02*
	15. How important do you consider personal care of your feet?	3.1	3.4	0.22	3.1	3.4	0.25	3.0	4.3	0.001**
	16. Regarding the recommendations on how to take care of your own feet, do you follow them?	3.0	3.3	0.24	2.8	3.5	0.08	2.9	4.3	0.002**
	Overall scores of the foot care practices	40.2	42.2	0.18	40.9	63.2	0.02*	36.2	73.1	0.001**

a Extracted using Wilcoxon test; *p < 0.05; **p < 0.01.

Footwear and socks practices followed a similar pattern, with Intervention Group 2 showing a notable increase from 11.3 to 23.2 (*p* = 0.01). However, both the control group and Intervention Group 1 exhibited non-significant changes (*p* = 0.19 and *p* = 0.25, respectively). Overall foot care practice scores further highlighted the intervention’s impact on Group 2, where scores significantly rose from 36.2 to 73.1 (*p* = 0.001). Although Intervention Group 1 showed a significant increase from 40.9 to 63.2 (*p* = 0.02), the control group did not experience a significant change (*p* = 0.18).

#### Changes in self-efficacy

Table 3 reveals limited progress in the self-efficacy subscale within the Control Group, with only three out of 12 items showing significant changes: protecting feet (*p* = 0.02), drying feet (*p* = 0.04), and routinely applying lotion to feet (*p* = 0.03). However, overall scores remained nearly unchanged (34.0 to 35.7; *p* = 0.21).

**Table 3:** Median scores for self-efficacy at baseline and 8-week follow-up within each group.

Outcome measures	Items	Control group		*p*-value^a^	Intervention group 1		*p*-value^a^	Intervention group 2		*p*-value^a^
		Baseline	Follow-up		Baseline	Follow-up		Baseline	Follow-up	
Self-efficacy	1. I can protect my feet	2.5	3.4	0.02*	2.8	3.7	0.04*	2.5	4.1	0.001**
	2. Even without pain/discomfort, I can look at my feet daily to check for cuts, scratches, blisters, redness or dryness	2.2	2.8	0.12	2.6	3.2	0.08	2.7	4.0	0.003**
	3. After washing my feet, I can dry between my toes	2.2	3.2	0.04*	2.7	3.8	0.02*	2.9	4.3	0.001**
	4. I can judge when my toenails need to be trimmed by a podiatrist	2.4	2.7	0.26	2.9	3.3	0.12	2.8	3.4	0.12
	5. I can trim my toenails straight across	2.7	3.0	0.07	2.8	3.2	0.18	3.0	4.2	0.002**
	6. I can figure out when to use a pumice stone to smooth corns and/or calluses on my feet	2.8	3.1	0.30	3.3	3.7	0.15	2.9	4.1	0.001**
	7. I can test the temperature of the water before putting my feet into it	2.6	3.0	0.11	3.1	3.8	0.04*	2.8	3.9	0.02*
	8. If I was told to do so, I can wear shoes and socks every time I walk (includes walking indoors)	2.5	2.9	0.18	2.6	3.4	0.03*	2.7	4.0	0.001**
	9. When I go shopping for new shoes, I can choose shoes that are good for my feet	2.4	2.8	0.22	2.7	3.3	0.07	3.2	3.8	0.05
	10. I can call my doctor about problems with my feet	2.3	2.9	0.13	3.2	3.7	0.25	2.7	4.1	0.03*
	11. Before putting them on, I can check the insides of my shoes for problems that could harm my feet	2.4	2.9	0.10	2.5	3.0	0.13	2.9	4.0	0.002**
	12. If directed to do so, I can routinely apply lotion to my feet	2.8	3.1	0.03*	3.0	3.8	0.04*	2.4	4.2	0.01*
	Overall scores of the self-efficacy	34.0	35.7	0.21	33.1	39.3	0.03*	34.5	48.2	0.001**

a Extracted using Wilcoxon test; *p < 0.05; **p < 0.01.

In contrast, significant improvements were observed in the overall self-efficacy scores for both Intervention Group 1 (33.1–39.3; *p* = 0.03) and Intervention Group 2 (34.5–48.2; *p* = 0.001). As outlined in Table 3, both intervention groups demonstrated significant improvements in self-efficacy, including protecting feet, drying feet after washing, consistently wearing shoes and socks when walking, and routinely applying lotion to feet.

#### Effects of the intervention on foot care practices

Table 4 shows that the intervention led to significant improvements in foot care practices, particularly in Intervention Group 2. In the unadjusted model, participants in this group demonstrated more substantial improvements in foot care practices compared to those in Intervention Group 1. The unadjusted OR for this comparison was 1.8 (95% CI: 1.1 to 3.0, *p* = 0.005), and the adjusted OR (aOR) was 1.7 (95% CI: 1.2 to 2.9, *p* = 0.02), indicating that the additional components of the intervention in Group 2 led to greater improvements in foot care practices compared to Group 1.

**Table 4:** GEE logistic regression analyzing the effect of the intervention on foot care practices and self-efficacy (baseline and follow-up).

Outcome measures	Unadjusted model		Adjusted model	
	OR (95% CI)	*p*-value	aOR (95% CI)	*p*-value
Foot care practices				
Intervention Group 1 vs. Control (Baseline)	1.5 (0.8–2.3)	0.18	1.3 (0.7–2.4)	0.25
Intervention Group 2 vs. Control (Baseline)	1.8 (1.0–3.4)	0.10	1.6 (0.9–3.1)	0.18
Intervention Group 1 vs. Intervention Group 2 (Baseline)	1.2 (0.7–2.1)	0.15	1.1 (0.6–2.0)	0.20
Group × Time Interaction (Intervention 2 vs. Intervention Group 1)	1.8 (1.1–3.0)	0.005**	1.7 (1.2–2.9)	0.02*
Group × Time Interaction (Intervention 2 vs. Control)	2.8 (1.4–5.6)	<0.001**	2.5 (1.3–5.1)	<0.001**
Group × Time Interaction (Intervention 1 vs. Control)	1.7 (0.9–3.2)	0.07	1.6 (0.8–3.0)	0.09
Self-efficacy				
Intervention Group 1 vs. Control (Baseline)	1.6 (0.9–2.8)	0.11	1.4 (0.8–2.6)	0.19
Intervention Group 2 vs. Control (Baseline)	2.0 (1.1–3.7)	0.05	1.9 (1.0–3.5)	0.06
Intervention Group 1 vs. Intervention Group 2 (Baseline)	1.3 (0.8–2.2)	0.14	1.2 (0.7–2.1)	0.18
Group × Time Interaction (Intervention 2 vs. Intervention Group 1)	2.0 (1.2–3.5)	0.003**	1.8 (1.1–3.2)	0.01*
Group × Time Interaction (Intervention 2 vs. Control)	3.0 (1.6–5.7)	<0.001**	2.7 (1.4–5.2)	<0.001**
Group × Time Interaction (Intervention 1 vs. Control)	1.8 (0.7–3.3)	0.05	1.7 (0.9–2.6)	0.08

*p < 0.05; **p < 0.01.

The final model adjusted for all participants’ characteristics listed in Table 1.

OR, odd ratio; aOR, adjusted odd ratio; CI, confidence interval.

“Intervention Group 1 vs. Control”: Indicates between-group differences at baseline between the Control Group and Intervention Group 1.

“Intervention Group 2 vs. Control”: Indicates between-group differences at baseline between the Control Group and Intervention Group 2.

“Intervention Group 1 vs. Intervention Group 2”: Indicates between-group differences at baseline between the Intervention Group 1 and Intervention Group 2.

Group × Time Interaction (Intervention 1 vs. Control): Indicates differences between Intervention Group 1 and the Control Group in the change of outcomes at 2 months relative to baseline.

Group × Time Interaction (Intervention 2 vs. Control): Indicates differences between Intervention Group 2 and the Control Group in the change of outcomes at 2 months relative to baseline.

Group × Time Interaction (Intervention 1 vs. Intervention 2): Indicates differences between Intervention Group 1 and the Intervention Group 2 in the change of outcomes at 2 months relative to baseline.

A comparison between Intervention Group 2 and the Control Group revealed the most significant improvements in foot care practices within Group 2. The unadjusted OR for this comparison was 2.8 (95% CI: 1.4 to 5.6, *p* < 0.001), and the aOR was 2.5 (95% CI: 1.3 to 5.1, *p* < 0.001). This underscores the superior effectiveness of the additional intervention components in Group 2 compared to those in the Control Group. Although Intervention Group 1 also showed improvements in foot care practices over the Control Group, the effects were less pronounced. The unadjusted OR for Intervention Group 1 versus the Control Group was 1.7 (95% CI: 0.9 to 3.2, *p* = 0.07), and the aOR was 1.6 (95% CI: 0.8 to 3.0, *p* = 0.09).

#### Effects of the intervention on self-efficacy

As detailed in Table 4, the intervention resulted in substantial improvements in self-efficacy, especially in Intervention Group 2. The interaction between group and time further highlighted that Intervention Group 2 experienced more substantial improvements over time compared to the Intervention Group 1, with an unadjusted OR of 2.0 (95% CI: 1.2 to 3.5, *p* = 0.003) and an aOR of 1.8 (95% CI: 1.1 to 3.2, *p* = 0.01).

Comparing Intervention Group 2 with the Control Group, the improvement in self-efficacy was the most notable. The unadjusted OR was 3.0 (95% CI: 1.6 to 5.7, *p* < 0.001), and the aOR was 2.7 (95% CI: 1.4 to 5.2, *p* < 0.001). Intervention Group 1 also experienced enhancements in self-efficacy relative to the Control Group, although these did not reach statistical significance. The unadjusted OR for this comparison was 1.8 (95% CI: 0.7 to 3.3, *p* = 0.05), and the aOR was 1.7 (95% CI: 0.9 to 2.6, *p* = 0.08).

## Discussion

This study evaluated the effectiveness of targeted educational interventions on foot care practices and self-efficacy among women with T2DM in northern Jordan. The findings indicate significant improvements in both areas among participants who received more interactive, tailored, and multifaceted educational approaches.

### Improved foot care practices

The study revealed that participants in Intervention Group 2 showed substantial improvements across several aspects of foot care, including personal care routines, podiatric care, and footwear choices. These findings suggest that comprehensive, multimodal educational approaches with interactive elements and targeted content are more effective at promoting positive behavioral changes than standard care. The passive nature of standard clinical care, which relies heavily on provider-directed instructions, often limits patient engagement, and reduces their ability to take control of their own health (53, 54). Furthermore, this model may overlook individual patient needs and preferences, resulting in a lack of understanding of preventive foot care and poor adherence to recommended practices (53, 54).

Although participants in Intervention Group 1, who received routine follow-up via text messages and calls, showed some improvement in foot care practices, the changes were less significant compared to those in Intervention Group 2. This disparity highlights the limitations of one-way communication methods, which often lack the depth needed to fully engage patients (55). Studies have shown that while reminders through messages or calls can prompt short-term behavioral changes, they often fail to provide the comprehensive education and hands-on training necessary for sustained improvements in self-care (49, 56). As a result, patients may struggle to fully integrate foot care practices into their routine, limiting their ability to reduce the risk of skin damage and prevent foot injuries and complications, such as ulcers and infections.

In Jordan, previous research has shown that gaps in knowledge and practices regarding foot care among diabetic women are largely due to insufficient exposure to tailored educational interventions (57, 58). Accordingly, developing strategies that provide practical, easy-to-understand instructions and involve family members in supporting foot care routines is crucial in culturally conservative settings like Jordan (59). Women, in particular, may face unique challenges in managing regular diabetic foot care due to factors such as limited access to healthcare, lower health literacy, and cultural norms (59, 60). This study’s focus on women underscores the importance of implementing gender-sensitive educational programs specifically designed to meet their needs and enhance their ability to address foot issues more effectively.

### Enhanced self-efficacy

In this study, participants in Intervention Group 2 demonstrated increased self-efficacy, showing greater confidence in performing foot care activities such as self-examinations, seeking medical attention, and completing essential tasks like daily foot inspections, applying lotion, and selecting appropriate footwear. These findings suggest that interactive educational approaches—incorporating group discussions, real-time problem-solving, and immediate feedback—can empower patients to take a more active role in managing their condition, thereby fostering competence in foot care practices. Our results align with other studies that highlight how improving self-efficacy is beneficial in enhancing knowledge, confidence, and promoting better foot health (56, 61, 62).

Our results also confirm that providing routine follow-up via text messages and calls led to an increase in self-efficacy within Intervention Group 1. However, compared to Intervention Group 2, the improvement was not as significant. This comparison suggests that routine reminders alone, while helpful in maintaining basic foot care behaviors, may fail to actively build a patient’s confidence to manage complex self-care activities independently (63). Our findings align with previous research, emphasizing the added value of dynamic experiences in improving foot health, such as hands-on learning, digital reinforcement tools, and opportunities for developing new skills in a supportive environment (63–65).

The gains in self-efficacy observed in Intervention Group 2, compared to the Control Group, also underscore the limitations of standard clinical care in equipping patients with the necessary tools to overcome obstacles, foster a sense of mastery, and reinforce their ability to execute foot self-care tasks independently. Currently, data on the quality of diabetic care in Jordanian health facilities is limited (66). Much of the research highlights a lack of meaningful patient engagement, particularly among women, with healthcare providers often relying on delivering general instructions rather than interactive, patient-centered approaches.

Diabetic women may face additional cultural and social obstacles that further discourage them from seeking foot care or participating in related activities, especially when male physicians are typically the primary care providers (67). The lack of female physicians can exacerbate these barriers by reducing effective communication, decreasing patient comfort, and hindering adherence to recommended care practices. To address these challenges, offering practical strategies through virtual sessions exclusively for women, led by female specialists, can improve the effectiveness of educational interventions. This approach creates more culturally sensitive, comfortable, and engaging environments, encouraging long-term adherence and proactive self-management.

### Management strategies and implications for practice

The study’s findings highlight several key management strategies and implications for clinical practice. These include:

### Multifaceted and tailored educational interventions

Obviously, the incorporation of tailored and multifaceted educational approaches engaging patients is crucial for improving diabetic foot care practices. Standard clinical visits may not fully support patients’ self-management, so incorporating a variety of educational methods—such as practical demonstrations, personalized digital content, and regular follow-ups—can significantly enhance patient engagement and adherence to foot care routines.

During clinical visits, healthcare providers can conduct routine assessments and risk stratification by categorizing DFD risk based on established guidelines. Implementing systematic screening protocols to identify patients at varying levels of risk allows for more personalized preventive strategies and interventions, ensuring that those at higher risk receive more intensive and targeted education and care.

### Incorporation of technology in patient education

With the increasing availability of mobile technology among Jordanians (68), leveraging mobile devices and online platforms to deliver educational content is an effective strategy to support diabetic foot care. Given the cultural sensitivities and social barriers some patients may face, healthcare providers can offer digital reminders, interactive content, and educational videos to reinforce learning and promote adherence to recommended foot care practices. This approach helps create a supportive and accessible environment for patients, making it easier to maintain consistent foot care, even outside the clinical settings. Furthermore, health educators must ensure that videos and other educational modalities are accessible and easily understood by the least educated and most vulnerable patients.

### Family involvement in foot care management

Strengthening family responsibilities in managing DFD can be a powerful strategy to enhance self-care and improve patient outcomes. This is particularly important for supporting vulnerable patients who are older adults, poorly educated, and have limited access to advanced technologies. By defining specific family roles in foot care and actively engaging them in the management process, challenges in self-care can be addressed more effectively. This approach fosters a supportive environment where families can help set lifestyle goals, such as preparing healthier meals, promoting regular exercise, and ensuring adherence to foot care routines. Families interested in participating in such initiatives may benefit from training to build their capacity in supporting expected self-care behaviors.

### Study strengths and limitations

The study has several notable strengths. First, it employed a pretest-posttest, quasi-experimental design, providing a robust framework for evaluating the effectiveness of educational interventions over time. This design offered valuable insights into how targeted strategies can improve foot care practices and self-efficacy among diabetic women. Additionally, the study was guided by the HBM, an evidence-based model that ensured the educational interventions were tailored to address specific barriers and motivators related to foot care, thereby enhancing their relevance and effectiveness. Furthermore, the study demonstrated cultural sensitivity by customizing educational materials to fit the specific context of Jordan, increasing both the engagement and applicability of the interventions for the target population.

However, the study had several limitations. The use of a nonprobability convenience sampling method limits the generalizability of the findings, as the sample may not fully represent the diversity of women with T2DM in Jordan. Furthermore, conducting the study in a single setting may further restrict the applicability of the results to other populations or settings. The relatively small final sample size due to participant attrition over the 8-week intervention period could affect the study’s statistical power, potentially impacting the ability to detect significant differences between groups. The short follow-up duration of 8 weeks may not have been sufficient to assess the long-term sustainability of the observed improvements in foot care practices and self-efficacy. Future studies should consider longer follow-up periods to evaluate the durability of intervention effects over time.

## Conclusion

The findings reveal that women with T2DM who received targeted interactive educational interventions showed the greatest improvements in both foot care practices and self-efficacy, compared to those who received standard clinical visits or routine follow-ups via text messages and calls without additional engaging components. This suggests that incorporating elements such as interprofessional collaboration, practical demonstrations, digital content, and family support is more effective in promoting better foot care behaviors and increasing patients’ confidence in managing their foot health.

## Data Availability

The raw data supporting the conclusions of this article will be made available by the authors, without undue reservation.
